# Rapid Determination of Cadmium Contamination in Lettuce Using Laser-Induced Breakdown Spectroscopy

**DOI:** 10.3390/molecules23112930

**Published:** 2018-11-09

**Authors:** Tingting Shen, Wenwen Kong, Fei Liu, Zhenghui Chen, Jingdong Yao, Wei Wang, Jiyu Peng, Huizhe Chen, Yong He

**Affiliations:** 1 College of Biosystems Engineering and Food Science, Zhejiang University, 866 Yuhangtang Road, Hangzhou 310058, China; shentingtingstt@163.com (T.S.); wwkong16@zafu.edu.cn (W.K.); zhenghuichen@zju.edu.cn (Z.C.); jdyao@zju.edu.cn (J.Y.); 15236193955@163.com (W.W.); jypeng@zju.edu.cn (J.P.); yhe@zju.edu.cn (Y.H.); 2 Key Laboratory of Spectroscopy Sensing, Ministry of Agriculture and Rural Affairs, Hangzhou 310058, China; 3 School of Information Engineering, Zhejiang A & F University, Hangzhou 311300, China; 4 State Key Laboratory of Rice Biology, China National Rice Research Institute, Hangzhou 310006, China; chenhuizhe@163.com

**Keywords:** cadmium contamination, lettuce, laser-induced breakdown spectroscopy, multivariate analysis, genetic algorithm

## Abstract

Quick access to cadmium (Cd) contamination in lettuce is important to supervise the leafy vegetable growth environment and market. This study aims to apply laser-induced breakdown spectroscopy (LIBS) technology for fast determination of Cd content and diagnosis of the Cd contamination degree in lettuce. Emission lines Cd II 214.44 nm, Cd II 226.50 nm, and Cd I 228.80 nm were selected to establish the univariate analysis model. Multivariate analysis including partial least squares (PLS) regression, was used to establish Cd content calibration models, and PLS model based on 22 variables selected by genetic algorithm (GA) obtained the best performance with correlation coefficient in the prediction set *Rp*^2^ = 0.9716, limit of detection (*LOD*) = 1.7 mg/kg. K-Nearest Neighbors (KNN) and random forest (RF) were used to analyze Cd contamination degree, and RF model obtained the correct classification rate of 100% in prediction set. The preliminary results indicate LIBS coupled with chemometrics could be used as a fast, efficient and low-cost method to assess Cd contamination in the vegetable industry.

## 1. Introduction

Toxic heavy metal cadmium (Cd) has become a common concern as it is ubiquitous in the environment and highly toxic for human [1]. Some lakes in industrialized areas in China have high Cd concentrations exceeding 0.8 μg/L [2], such as Luan river with Cd concentrations 1.120–4.474 μg/L and East Lake which is close to 8 μg/L [3]. Soil also have an excessive accumulation of Cd due to some human activities, such as the release of waste, usage of chemical fertilizers, pesticides, and sewage sludge in agricultural lands [4]. Cd pollution in water, soil, and other environments has increased the Cd accumulation possibility in food, such as vegetables and cereals. Then, Cd easily enters the human body through the food chain and increases the risk of cancer, mutation, endocrine disorder, renal failure, and chronic anemia for human [5].

Lettuce (*Lactuca sativa L. var. longifolia*) is a plant species produced and consumed worldwide. As a common leafy vegetable, lettuce is rich in fiber, vitamins (A, B, C, and K), chlorophyll, and carotenoids which are vital for human health [6]. Nevertheless, it has been reported that lettuce has a comparatively high accumulation of cadmium in its leaves [7], and it has been proposed as an indicator plant to test cadmium polluted soils and plant tissues [8]. The characteristic of high Cd accumulation in lettuce leaves, unquestionably, increases human dietary risk. Heavy metals in food are hidden and irreversible. Due to the advantages of a high accumulation of Cd, determination of lettuce cadmium levels is beneficial for judging whether lettuce is a healthy food, supervising the growth environment of vegetables, and decreasing the risk associated with Cd toxicity.

Atomic absorption spectrometry (AAS), inductively coupled plasma optical emission spectroscopy (ICP-OES), and inductively coupled plasma with mass spectrometry (ICP-MS) are common methods to detect concentrations of heavy metal Cd in food [9]. Though the results are accurate, these traditional methods are expensive and need complex sample preparation with a strongly acidic environment and high temperatures for sample digestion [10]. This process is time-consuming. Obviously, the above deficiencies limit the potential of real-time monitoring and rapid detection of heavy metals which are highly desirable in regulating heavy metal pollution in the lettuce market and for efficient lettuce growth.

Laser-induced breakdown spectroscopy (LIBS) is a recently developed elemental analytical technique that has a fast responsive, micro-destructive and chemical-free with no or little sample preparation [10]. Given these merits, LIBS has been widely used in different food detection methods, such as meat classification [11], Cu determination in fruits [12], Na determination in bakery products [13], and bacterial pathogen determination in milk [14]. Studying the rapid detection of heavy metals in plants is conducive to quickly determining concealed characteristic of heavy metal pollution in food, providing a decision-making basis for early and rapid diagnosis of heavy metal pollution, and is capable of carrying out targeted measures to cut off the accumulation of heavy metal pollution and its irreversible damage for lettuce safety. Recently, there have also been some reports focusing on heavy metal detection in plants. Yao et al. successively mixed cabbages with lead [15] and cadmium [16], then used LIBS to detect the heavy metals. The high determination coefficient and low root mean square error (RMSE) of the detection models indicate it is feasible to use LIBS to determine heavy metals in leafy vegetables. However, the uniformity of mixing is a concern and the sample size needs to be improved. To get close to the actual plant-environment system and reflect the heavy metals in plant tissues, some plants under different heavy metal stresses, are better planted. Chromium content in rice leaves [17] and copper content in Tobacco leaves [18] were detected by LIBS technology, and good detection models were obtained. To our best knowledge, Cd analysis in lettuce—high Cd accumulation plant-based—with LIBS has not been investigated.

In this study, we focused on Cd fast analysis in lettuce based on LIBS technology and chemometrics, including Cd concentration fast detection and Cd pollution level fast discrimination. The specific objectives of this study were (1) to preprocess raw LIBS spectra by the autoscaling method, and to investigate univariate analysis based on Cd emission lines and obtain the most sensitive Cd emission line; (2) to compare multivariate analyses based on full spectra, Cd emission bands, and discontinuous variables selected by genetic algorithm (GA) and obtain the best and stable partial least squares (PLS) regression model to quickly detect Cd content; (3) to apply principal component analysis (PCA) to show the Cd pollution level in a three-dimensional perspective, and to use K-Nearest Neighbors (KNN) and random forest (RF) based on full spectra and variables selected by PCA loadings to classify Cd contamination level of lettuce.

## 2. Materials and Methods 

### 2.1. Sample Preparation

Lettuce (*Lactuca sativa* *L. var. longifolia*) leaves with different degrees of cadmium stress were used in this experiment. Romaine lettuce seeds were purchased from Qingxian Chunfeng Vegetable Variety Breeding Farm (Cangzhou, Hebei, China). The hydroponic experiment was carried out on Zijingang Campus, Zhejiang University, Hangzhou, China. The seeds were sterilized with 1% NaClO solution for 25 min, rinsed with sterile distilled water, and then germinated on sterile Murashige and Skoog culture medium at 35 °C and 65% relative humidity for 5 days. The seedlings with root length of approximately 3 cm were transplanted into 10 L full strength Yamazaki’s nutrient solution [19], and the culture solutions were renewed every 3 days. Growth conditions [20] were adjusted to 27/22 °C (16:8 h light-dark cycle), 65% relative humidity and a light intensity of 200 μmol m^−2^ s^−1^. After 9 days, 5 different cadmium treatments (0, 10, 30, 60, and 100 μM cadmium prepared by CdCl_2_ solution) were adopted in this experiment with similar size plants. The cultivation process of lettuce samples is shown in Appendix A. On the 30th day after treatment, leaves of each plant lettuce were cleaned by distilled water, dried at 60 °C for 5 h in an oven, and ground to powder separately. The cultivation process of lettuce samples and the lettuce leaves after 30 days of different Cd stress treatment showed no differences with visual observation. One hundred and fifty milligrams of single lettuce powders were pressed into a square pellet by a tablet pressing machine (FY-24, SCJS, Tianjin, China) with a pressure of 600 MPa for 30 s.

### 2.2. LIBS Measurements

**Figure 1:**
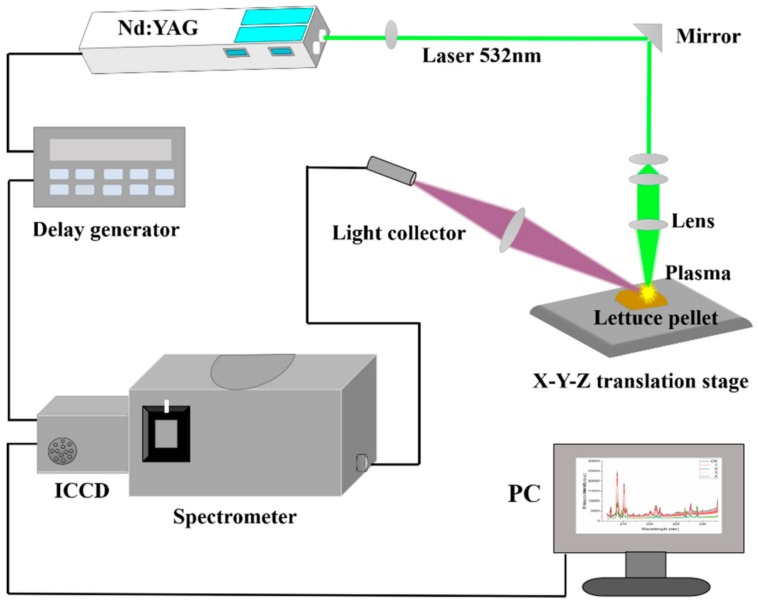
Schematic diagram of the laser-induced breakdown spectroscopy (LIBS) experimental setup.

The self-assembled LIBS device used in this experiment is presented in Figure 1. Combined with previous research [17], Q-switched Nd:YAG pulse laser (Vlite 200, Beamtech, Beijing, China) was used to generate laser pulses at 532 nm with a maximum energy of 200 mJ and 8 ns pulse width. After passing through our self-made optical system, the laser was finally focused on the sample surface through a plano-convex lens (*f* = 100 mm). After the laser ablated the sample mass, plasma was generated and diffused outward to emit electromagnetic waves. The waves were collected by a light collector and received by a spectrometer (SR-500i-A-R, Andor Technology, Belfast, UK) combined with an intensified charge coupled device (ICCD) camera (DH334T-18F-03, Andor Technology, Belfast, UK), and spectra between 211.92 nm–232.90 nm with 0.02 nm resolution was collected. A delay generator (DG645, Stanford Research Systems, Sunnyvale, CA, USA) was used to control the delay time between the ICCD camera and laser Q-switch. Before the experiment, we optimized the experimental parameters and obtained optimal parameters with a laser energy of 60 mJ, delay time of 1.5 μs, and gate width of 10 μs. An automatic x–y–z translation was used to place lettuce pellets and control the laser ablation path with 4 × 4 array craters and each crater had 5 times the accumulation of laser pulses. To reduce fluctuation between the laser point-to-point, the spectrum for each sample was recorded by an average of the 80 spectra (4 × 4 × 5). Time of LIBS information collection for one sample was about 1min.

### 2.3. Detection of Lettuce’ Cd Content by AAS

Each pellet after LIBS acquisition was weighed and added into a Modified polytetrafluoroethylene vessel and mixed with 5 mL of 65% HNO_3_ and 1 mL of 30% H_2_O_2_ for microwave digestion. The digested liquid was diluted to a volume of 30 mL with high-purity water by a weighing method after acid elimination at high temperatures. The reference Cd contents of the solution were determined with a flame atomic absorption spectrophotometer (AAS) (AA800, PerkinElmer, Waltham, MA, USA). Standard material, GBW10020 (Beijing, China), was used to control the analysis quality. The above pretreatment to obtain the solution for AAS needed more than 150 min. The reference cadmium values, which are statistically significant, accumulated in the 70 lettuce leaves and are shown in Table 1. As shown in Table 1, the mean Cd content of each group shows that the accumulation of cadmium in lettuce increases with the increase of cadmium stress even if not linearly. 

### 2.4. Data Analysis

Autoscaling method, also called normalized standard score method, could eliminate the magnitude influence of different variables [21] and reduce random noise from instruments and the experimental environment. The principle is to generate a normalized standard score by finding the normal curve *Z-score* equivalent for a given percentile rank, then transforming this *Z-score* to a score representing a distribution having a specified mean and standard deviation [22]. The formula is as follows:

(1) Z-score=(X−mean(X))/Std(X)

For spectral matrix *X*, the *Z-score* is computed using the mean and standard deviation along each column of *X.*

As one of the most important branches of intelligent computing, genetic algorithm (GA) has the characteristics of stronger robustness, global rand search, and the ability to find the global optimal solution in complex, multi-peak large solution spaces [23]. The calculations consist of the following steps [24]: Creating the initial population; sorting chromosomes and retaining the top hall; recombining, breeding, and mutating until achieving the maximum number of generations; bringing in an independent test set to find the top best chromosomes. A chromosome represents a bit vector, and the size of the chromosome population is defined by the number of chromosome-variables.

K-Nearest Neighbors (KNN) is based on the idea that the category of a data point is determined according to the classification of the nearest k [25]. In reality, the *k*-value is usually an odd number and defines the locality of KNN [26]. A *k*-value was chosen for optimal results with a minimum prediction error. 

Random forest (RF) is an ensembled learning technique and has the merit of being correct for decision trees’ tendency to overfit their training data [27,28]. For RF, the operation is as follows: n tree bootstrap samples are drawn from the calibration set and stored as a new set; each bootstrap sample grew an unpruned regression tree, and each node of the tree chose the best split among those variables; predict majority voted for classification by aggregating the predictions of the n trees.

### 2.5. Performance Evaluation

For Cd content quantitative detection, root mean square cross validation error of calibration set (RMSECV) and root mean square error of prediction set (RMSEP) suggest the accuracy of the calibration and prediction model, respectively. The correlation coefficient (*R*^2^) between reference Cd content and measured Cd content by the quantitative model was used to evaluate the detectability of different variables; sensitivity informs what fraction of the analytical signal is due to the increase of the concentration of a particular analyte at unit concentration [29]. In multivariable PLS calibration models, sensitivity is defined as:

(2) $$SEN=1/\left\|b_{k}\right\|$$

where *b_k_* is the regression coefficients of PLS calibration model, means the Euclidean norm of the *b_k_* vector.

In addition, limit of detection (*LOD*) was used to evaluate the sensitivity of univariate calibration and is expressed as:

(3) LOD=3s/b

where *s* is the standard deviation of the background intensities and *b* is the slope of the calibration curve.

*LOD* in the multivariate domain has recently been discussed in several multivariate techniques [30,31] and an approximation *LOD* of multivariable PLS calibration models can be estimated by combining Equations (2) and (3) [32,33]

(4) $$LOD=3s(1/SEN)=3s\left\|b_{k}\right\|$$

for qualitative determination of cadmium pollution degree, accuracy of classified rate was used to demonstrate the results [34].

### 2.6. Software Tools

LIBS spectra acquisition was carried out by Andor SOLIS for Imaging (v4.26, Andor Technology, Belfast, UK). Data analysis was executed by MATLAB R2017a (The MathWorks, Inc., Natick, MA, USA). Origin Pro 2015 (Origin Lab Corporation, Northampton, MA, USA) was applied for graphs designing.

## 3. Results and Discussion

### 3.1. Spectra Analysis

**Figure 2:**
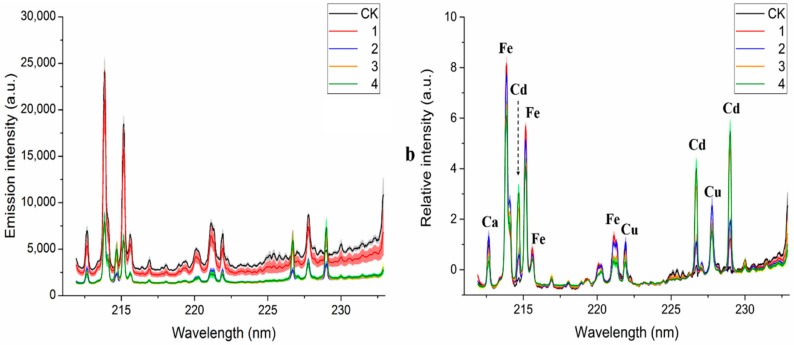
Average ± standard deviation plot of (**a**) original spectra and (**b**) those after autoscaling method pre-treatment.

The average raw LIBS spectra (line) and standard deviation (shadow on the line) profiles of the five different Cd-stress group lettuces are shown in Figure 2a. LIBS spectra of the five Cd-stress groups show similar tendencies which indicate that the samples contained similar elementary compositions and matrices. System instability and environmental fluctuations would cause unnecessary information redundancy and random errors on the acquired spectra. Therefore, autoscaling method was used to reduce random errors and correct baselines, and the preprocessed spectra are displayed in Figure 2b. All spectra remained the in the same dimension and background baseline. 

Based on the Kurucz database and the National Institute of Standards and Technology (NIST) Atomic Spectra Database (ASD), three Cd emission lines (ionic emission lines Cd II 214.44 nm and Cd II 226.50 nm, atomic emission lines Cd I 228.80 nm) were observed in all Cd stress lettuce samples. We also found that ionic emission lines Fe II 213.70 nm, Fe II 214.93 nm, and Cu II 227.62 nm all emerged near the cadmium emission lines. This phenomenon indicated the micronutrients Fe and Cu belonged to the matrix atoms of lettuce leaves and had a similar stimulated absorption energy with Cd. As Figure 2b shows, the intensity of the same element in different Cd-stress treatment has obvious differences which are not shown in Figure 2a, such as Fe emission lines in Group 1 have the highest intensity, and Cd emission lines in Group 4 have the highest intensity. Those differences indicated that heavy metal Cd stress significantly changed the content of elements in lettuce leaves.

### 3.2. Cd Content Prediction

The LIBS spectra after the autoscaling method were evaluated for Cd content of lettuce samples by univariate and multivariate data analysis with the partial least squares (PLS) regression method. Before quantitative analysis, 70 samples were partitioned into a calibration set (47 samples) and a prediction set (23 samples) based on Kennard-Stone (KS) algorithm which could avoid bias in sample selection [35]. 

#### 3.2.1. Univariate Analysis

**Figure 3:**
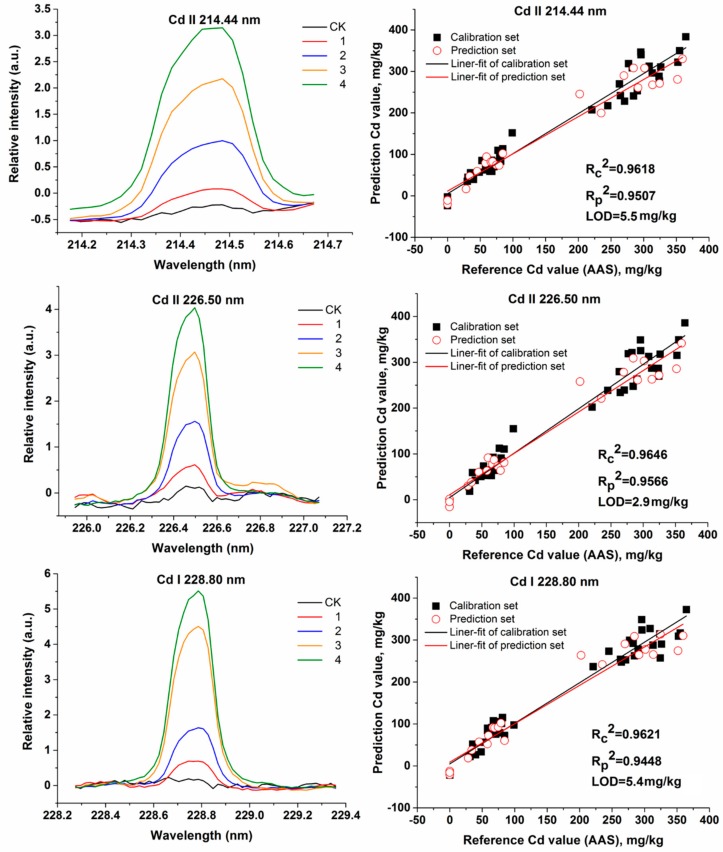
Cd II 214.44 nm, Cd II 226.50 nm, and Cd I 228.80 nm peaks and corresponding univariate analysis curve fitting plots.

As a traditional calibration method, univariate analysis for LIBS analysis relates spectral intensities of only one emission line with reference element content values to generate a calibration curve. Figure 3 presents the average spectrum of each group lettuce samples in Cd II 214.44 nm, Cd II 226.50 nm, and Cd I 228.80 nm, respectively. As shown in Figure 3, there is no self-absorption or interruption of other emissions in the three Cd emission lines. So, the spectral intensities of the three Cd emission lines were used as input variables for univariate analysis, respectively, and these three lines were also discussed in the report of Yao et al. [16]. 

Figure 3 shows univariable calibration results based on Cd II 214.44 nm, Cd II 226.50 nm, and Cd I 228.80 nm, respectively. The three univariable calibrations all demonstrate the predictive capability with *Rp*^2^ value of more than 0.94 and *LOD*s of less than 5.5 mg/kg. The results also indicate that the peak intensity of emission line 226.50 nm has the best capability (*Rc*^2^ = 0.9646, RMSECV = 23.9 mg/kg, *Rp*^2^ = 0.9566, RMSEP = 27.4 mg/kg, *LOD* = 2.9 mg/kg) to predict cadmium content, which obtained higher *R*^2^ values and lower RMSEs, and *LOD*s in all univariate calibration models. Univariate analysis based on the above Cd emission lines are more beneficial for exploiting a portable instrument for rapid detection of heavy metal Cd in Lettuce Market, undoubtedly.

#### 3.2.2. Multivariable Analysis

As an effective calibration method, multivariate analysis utilizes more useful spectral information to analyze the relationship between LIBS spectra and Cd content in lettuce leaves. PLS regression was tried to establish calibration models, and full-cross validation was performed to avoid overfitting [36,37]. The number of latent variables (LVs) was optimized in the calibration model for all the PLS models. The range of full LIBS spectra from 211.70 nm to 232.68 nm with 2014 variables were selected to construct the PLS model. The whole peak band of each strong Cd emission line—the range of 214.17–214.67 nm, 225.94–227.07 nm, 228.27–229.35 nm—was also used as the input for the PLS model, respectively. We combined the intensity of Cd II 214.44 nm, Cd II 226.50 nm, and Cd I 228.80 nm and also used GA to select the most relevant variables from the full spectra to improve PLS model after 1000 iterations. With a decrease of 46.5 times, the 22 variables selected by GA are shown in Table 2. Most variables in the 22 variables were located at the left and right sides of the three strong Cd emission lines, such as 226.42 nm, 226.44 nm, 226.46 nm, 226.54 nm, and 226.56 nm which were in the near vicinity of Cd II 226.50 nm; 228.64 nm, 228.74 nm, 228.72 nm, 228.66 nm, 228.68 nm, 228.76 nm, 228.80 nm, 228.78 nm were in the near vicinity of Cd II 228.80 nm, and 214.44 nm, 214.36 nm, 214.48 nm, 214.58 were in the near vicinity of Cd II 214.44 nm. In total, the above 18 variables belonged to part of Cd information in LIBS spectra. Among the 22 variables, 222.23 nm, 222.25 nm, 222.27 nm, 230.94 nm represented LIBS background information. After a thousand iterations, the background information still preserved, and this phenomenon indicated that the four variables might have a unique relationship with the lettuce matrix and could explain lettuce matrix information in PLS calibration model.

**Figure 4:**
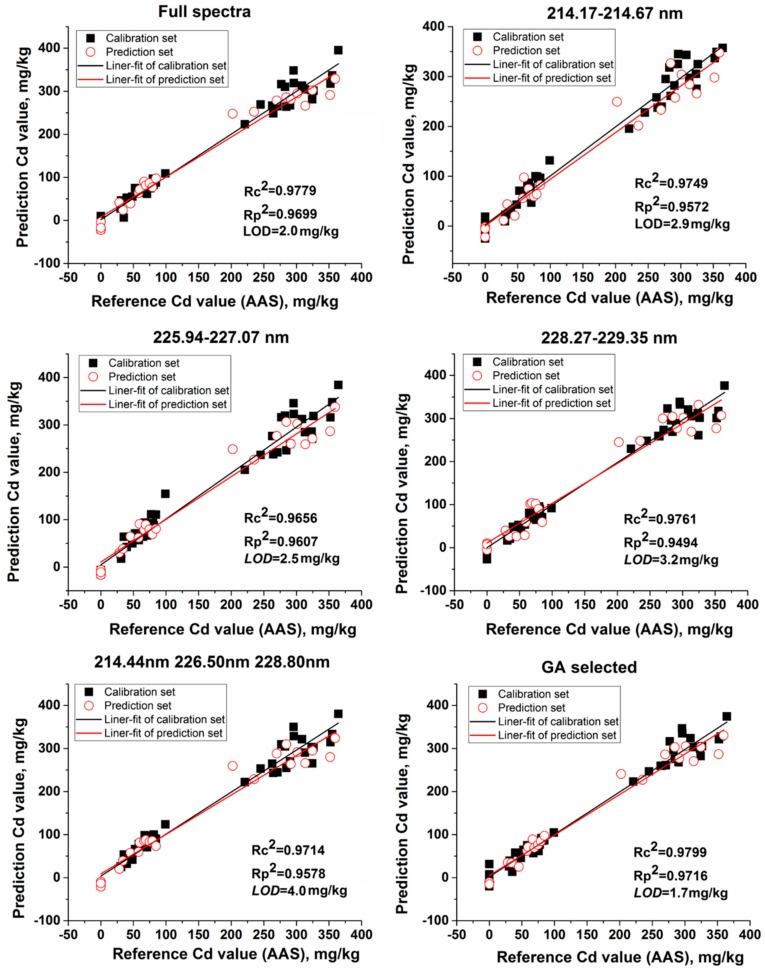
The relationship between reference Cd value and LIBS measured Cd value that predicted by partial least squares (PLS) regression models based on different variables.

The results for the multivariate analysis by PLS regression with different variables are shown in Appendix B and Figure 4 shows the scatter plots of the models. As Figure 4 shows, all the PLS models achieved good performance in the calibration set and prediction set, with *Rc*^2^ and *Rp*^2^ higher than 0.9494. The model based on Cd peaks 214.39–214.89 nm, 226.15–227.29 nm, and 228.49–229.57 nm were all better than the univariable analysis based on the intensity of single wavelength (Cd II 214.44 nm, Cd II 226.50 nm, or Cd I 228.80 nm). This is because Cd peaks did not only contain features near the three strong Cd emission lines but also contained some background information and matrix information which had relevance to the substrate of the lettuces. The PLS model of full spectra had better results with *Rc*^2^ of 0.9779 and *Rp*^2^ of 0.9699 because full LIBS spectral contained all emission lines for elements and continuous background information. But full LIBS spectra inevitably introduced noise or irrelevant information which resulted in model complexity and instability [38,39]. GA was used to screen the 22 most effective variables associated with Cd content and obtained the best result in the calibration set with *Rc*^2^ = 0.9799 and the prediction set with *Rp*^2^ = 0.9716, and the *LOD* of PLS model was 1.7 mg/kg. 

Compared with univariate analysis, all models for multivariable analysis were found to reasonably fit because of the merit of combining useful multi-variables to deal with matrix effect and shot-to-shot fluctuation of the LIBS spectra. PLS regression could correlate the maximal variance in independent variables (LIBS variables) with the dependent variable (Cd values by AAS) using the regression method and reduced multicollinearity of independent variables [36]. Genetic algorithm selected the effective variables through thousands of iterations, so the screened variables were representative for Cd content in lettuce leaves. Therefore, multivariate analysis combining with GA-PLS is more suitable for accurate detection of Cd content in lettuce leaves for rigorous laboratory research and food market regulation. Compared with univariate analysis, the calibration model based on the three Cd emission lines is more beneficial for fast detection in the lettuce growth source and market.

### 3.3. Cd Pollution Degree Analysis

Under the stress of Cd pollution, concentrations of some elements in lettuce leaves changed gradually. As a fingerprinting atomic spectroscopy, LIBS could capture these variations in elements. It was difficult to distinguish Cd pollution degree of lettuce leaves by visual observation, so LIBS, combined with chemometrics, was applied to solve this problem.

#### 3.3.1. PCA

PCA converted the original LIBS variables into new variables (PCs) so that a few new variables (PCs) were linear combinations of the original LIBS variables. These PCs were orthogonal and unrelated to each other, eliminating the possible multicollinearity between the original variables. In general, the first few PCs interpreted the most useful data and could be applied to observe the distribution of the samples and identify the differences between them visually [40,41]. So, principal component analysis (PCA) was applied to display the classification trend of lettuce samples in three-dimensional principal components (PCs) plot first. 

**Figure 5:**
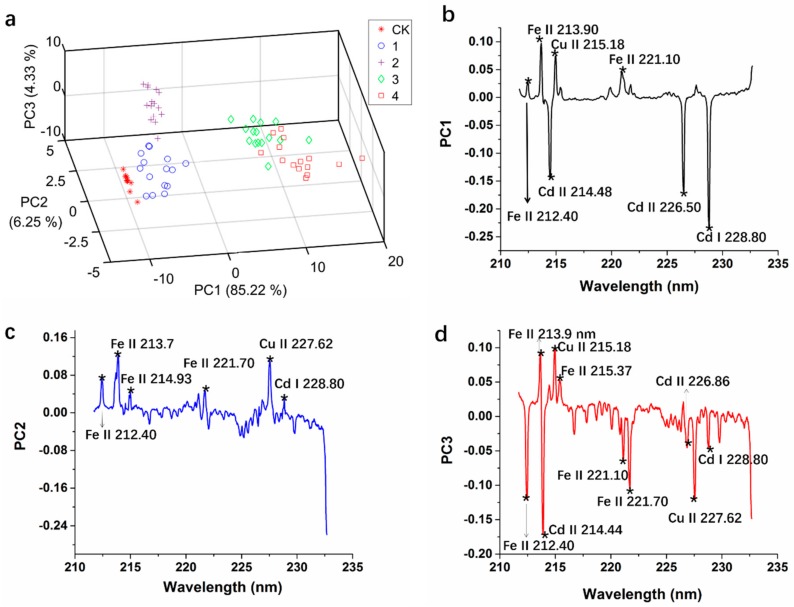
Three-dimensional principal components plot (**a**) of for different Cd pollution groups of lettuce leaves, the first three principal component analysis (PCA) loadings plot (**b**–**d**) with the variables selected by PCA.

All LIBS spectra for principal component analysis were pretreated by the autoscaling method. The accumulated variance contribution rate was up to 95.8% LIBS raw variables of lettuce leaf samples for the first three PCs. Figure 5a shows score scatter plots of the first three PCs with PC1, PC2, PC3 and explains 85.2%, 6.25%, 4.33% of total LIBS variables, respectively. The Cd^2+^ group 0 μM, 10 μM, and 30 μM were close but completely separated from each other. The samples of group 0 μM distributed closely which meant there was almost no difference between lettuce leaves in group 0 μM. The Cd^2+^ group 60 μM and 100 μM were far away from other groups, and there was overlap between these two groups. The good separation signified obvious differences of lettuce leaves between the different Cd pollution degrees, while the overlaps may come from the obstruction of similar leaf matrix ingredients. The Cd^2+^ group 60 μM and 100 μM belonged to more severe Cd pollution and presented some similar matrix components.

PCA loadings are the coefficients of the original variables on the PCs and reflect the degree of correlation between the principal component and the original wavelength variable of the spectrum. A greater absolute value of one loading indicates its corresponding LIBS raw variable contains more useful and important information. The first three PCs expressed 95.8% LIBS raw variables, and the first three PCA loadings are plotted in Figure 5b,c,d. The variables with an absolute value of loading larger than 0.04 were selected as the important variable for LIBS spectra of lettuce leaves with Cd pollution. As Figure 5 shows, 16 variables were selected by PCA loadings and represent the important and characteristic LIBS spectral information for lettuce leaf matrix with Cd pollution. The three Cd emission lines and the variables which belonged to the Cd peaks (226.86 nm and 214.48 nm) were screened, and some emission lines for Iron (Fe) and copper (Cu) also belonged to high contribution rate variables. Fe and Cu are important nutrients in the growth of lettuce, and different Cd stress forced absorption differences of these important elements in lettuce leaves. 

#### 3.3.2. Classification Models for Cd Pollution Degree

A three-dimensional principal components plot showed the distribution trends of lettuce samples and could not define the boundaries of each category definitely, so classification methods were used for discriminating samples in different Cd pollution degrees. KNN and RF were applied to establish the classification models. Three kinds of variables, including raw full spectra, full spectra after autoscaling method (*Z-score*) and the 16 important variables selected by PCA loadings, were input to the classification models and compared to obtain stable and efficient classification models. KNN models were built with k ranging from 3 to 10, and the best results were obtained with a k of three for the three kinds of variables as shown in Table 3. For RF models, the number of regression trees in the forest was optimized from 50 to 200 with a step size of one and nodes per tree were optimized from 1 to 50 with a step size of five. 

For raw full spectra, the KNN model and the RF model obtained unpleasant results with correct recognition rates of 84.6% and 80.0% in prediction set, respectively. The reason for the poor performance was that the original spectra contained many random errors such as environmental differences, instrument noise, and background noise, etc. After the autoscaling preprocesssing, the full spectra performed better with 95.5% correct recognition rates in the prediction set for the KNN model and 100% for the RF model. After removing random noise, the spectral information purely expressed the similarities and differences of lettuce ingredients. Therefore, the optimal classification models for classifying Cd contamination level of lettuce is the RF model (68 trees and 25 nodes per tree) based on the *Z-score* full spectra with correct recognition rates 100% and 100% in the calibration and prediction set as shown in Table 3. The RF model based on *Z-score* full LIBS spectra performed better than the KNN model, because RF could deal with irrelevant features and assign data features to different weights through the order of branch bifurcation.

As shown in Table 3, all models of variables screened by PCA loadings obtained acceptable results with classification accuracies over 92.0%. RF classification model (50 trees and 1 node per tree) based on 16 optimal emission lines obtained the best performance, with the classification rate of 100% in the calibration set and the classification rate of 96.0% in the prediction set. The results indicated that it is feasible to fast detect Cd pollution degree on lettuce leaves using an accurate single model based on the 16 optimal variables selected from the LIBS spectra. Another advantage is that the number of input variables reduced from 1024 to 16, leading to a reduction of 98.4%. However, the PCA loading selection method still loses a small amount of LIBS information so the recognition rate of the prediction set could reach 100%, and more variable screening methods can be tried subsequently.

## 4. Conclusions

In this experiment, we have shown the potential of rapid analysis of heavy metal cadmium contamination in lettuce using laser-induced breakdown spectroscopy. The rapid analysis focused on the fast detection of Cd content and the diagnosis of Cd contamination levels of lettuce leaf samples with accurate results. A total of three Cd emission lines Cd II 214.44 nm, Cd II 226.50 nm, and Cd I 228.80 nm were selected to establish univariate analysis model, and the intensity of Cd II 226.50 nm performed the best with *Rc*^2^ value of 0.9646, *Rp*^2^ value of 0.9566, and *LOD* = 2.9 mg/kg. For multivariable analysis, all PLS models based on six variables achieved better performance than univariable analysis, with *Rc*^2^ and *Rp*^2^ higher than 0.9494. The best prediction result was achieved by GA-PLS model based on 22 variables with *Rc*^2^ = 0.9799, *Rp*^2^ = 0.9716, and the *LOD* of PLS model was 1.7 mg/kg. For the 22 variables, there were 18 variables featured on left and right sides for the three strong Cd emission lines and four variables represented in the LIBS background. KNN and RF models based on raw LIBS spectra, *Z-score* LIBS full spectra, and variables selected by PCA loadings were established to rapidly diagnose Cd contamination levels of lettuce leaves. The RF model (68 trees and 25 nodes per tree) based on *Z-score* spectra performed the best with correct recognition rates of 100% in both the calibration and prediction set. The RF model of variables selected by PCA loadings also obtained acceptable results with a prediction accuracy of 96.0% with input variables reducing from 1024 to 16.

The proposed approach provides a fast, simple, and precise method for effective quantitative and qualitative detection of heavy metal Cd contamination in biological samples of lettuce leaves by LIBS technology based on the appropriate chemometric methods. In addition, the proposed three Cd emission lines are available for the development of a portable instrument to detect Cd contamination in the vegetable market. However, based on our study, further advances are still needed. More samples with other chemometric methods to detect heavy metals in plant and growth environments, such as soils, water and gas, should be quickly explored for safe growth environment regulation to ultimately provide a fast and accurate technique for regulation and relief of heavy metal pollution in the food market.

## Figures and Tables

**Table 1:** Cadmium (Cd) content of lettuce leaves obtained by atomic absorption spectrophotometer (AAS) (mg/kg).

Groups	CK	1	2	3	4
Number	10	15	15	15	15
Min	0	28.4	58.2	202	221
Max	0.004	81.3	98.9	352	492
Mean	0.001	43.9	69.8	287	318
S.D.	0.003	22.8	7.14	37.3	64.0

Note: Group CK means the group for control check (CK) and represents 0 µM Cd stress; Group 1 represents 10 µM Cd stress; Group 2 represents 30 µM Cd stress; Group3 represents 60 µM Cd stress; Group 4 represents 100 µM Cd stress. These expressions apply to the full text. “S.D.” means standard deviation.

**Table 2:** The detail of 22 variables selected by genetic algorithm (GA) based on National Institute of Standards and Technology (NIST) database.

Elements	Emission Line (nm)	Selected Variables (nm)
Cd I	228.80	228.64, 228.74, 228.72, 228.66, 228.68, 228.76, 228.80, 228.78
Cd II	226.50	226.44, 226.42, 226.56, 226.50, 226.46, 226.54
Cd II	214.44	214.44, 214.36, 214.48, 214.58
background	/	222.23, 222.25, 222.27, 230.94

**Table 3:** Results of classification models using different variables.

Variables (Number)	Number	Model	Parameter ^[a]^	Calibration Set (%)	Prediction Set (%)
Raw spectra	1024	KNN	3	91.1	84.6
		RF	(58, 5)	100	80.0
*Z-score* Spectra	1024	KNN	3	96.0	95.6
		RF	(68, 25)	100	100
PCA selected	16	KNN	3	93.3	92.0
		RF	(50, 1)	100	96.0

^[a]^ Parameter means the parameters of the models: k value of K-Nearest Neighbors (KNN), number of trees in the forest and nodes per tree for random forest (RF).

**Figure A1:**
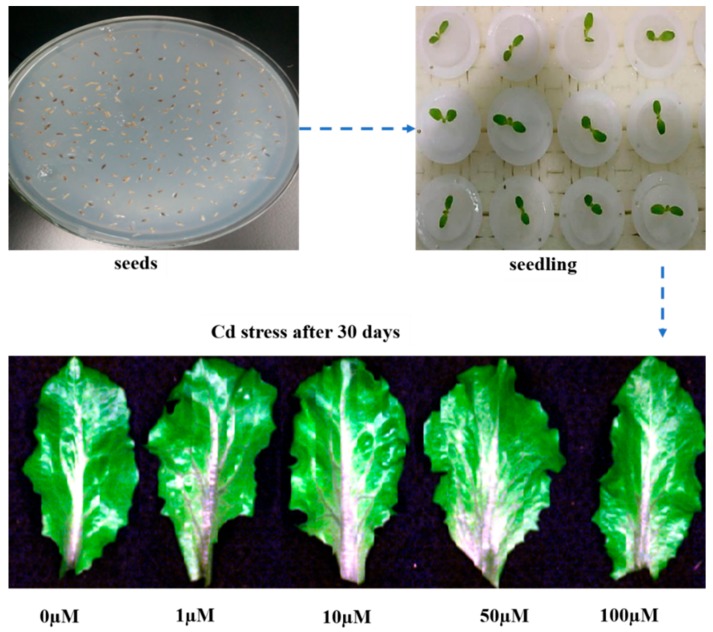
The cultivation process of lettuce samples in different cadmium treatments (0, 10, 30, 60, 100 μM).

## Appendix B

**Table A1:** **Table****A1.** The results for multivariate analysis by PLS regression with different variables.

Variables	Number	LVs	*LOD* mg/kg	Calibration Set		Prediction Set	
				*R_c_* ^2^	RMSECV mg/kg	*R_p_* ^2^	RMSEP mg/kg
Full Spectra	1024	4	2.0	0.9779	19.2	0.9699	23.1
214.17–214.67 nm	25	5	2.9	0.9749	20.4	0.9572	28.3
225.94–227.07 nm	56	4	2.5	0.9656	23.8	0.9607	27.0
228.27–229.35 nm	54	4	3.2	0.9761	19.9	0.9494	29.0
214.44 nm, 226.50 nm, 228.80 nm	3	1	4.0	0.9714	21.7	0.9578	27.2
GA Selected	22	5	1.7	0.9799	18.2	0.9716	22.4
